# Neurological complication burden and in-hospital mortality in non-HIV tuberculous meningitis across the lifespan: a retrospective cohort study

**DOI:** 10.3389/fmed.2026.1895708

**Published:** 2026-09-09

**Authors:** Yanhua Zhou, Qiong Wu, Xiangzhi Xiao, Huashan Zhou, Yan Ouyang, Jue Hu, Zhen Wang, Wengao Zeng

**Affiliations:** 1 Department of Otorhinolaryngology, The Second Hospital of Changsha (West Branch of Changsha Hospital for Maternal & Child Health Care), Changsha, Hunan, China; 2 Department of Neurosurgery, The Affiliated Changsha Central Hospital, Hengyang Medical School, University of South China, Changsha, Hunan, China; 3 Department of Neurology, The Second Hospital of Changsha (West Branch of Changsha Hospital for Maternal & Child Health Care), Changsha, Hunan, China; 4 Department of Pathology, The Second Hospital of Changsha (West Branch of Changsha Hospital for Maternal & Child Health Care), Changsha, Hunan, China; 5 Department of Neurology, The Affiliated Changsha Central Hospital, Hengyang Medical School, University of South China, Changsha, Hunan, China

**Keywords:** decision curve analysis, seizures, hydrocephalus, in-hospital mortality, intracranial pressure, neurological complications, tuberculous meningitis

## Abstract

**Background:**

Tuberculous meningitis (TBM) is a severe form of extrapulmonary tuberculosis and is frequently complicated by neurological injury. Existing studies have usually evaluated individual complications or admission-based prognostic models, whereas cumulative neurological complication burden documented across a hospitalization remains poorly characterized in non-human immunodeficiency virus (HIV) cohorts spanning pediatric and adult patients. We evaluated whether neurological complications present at admission or documented later during the index hospitalization were associated with in-hospital mortality.

**Methods:**

We conducted a single-center retrospective cohort study of non-HIV TBM patients hospitalized between October 1, 2013, and January 1, 2024. Potentially eligible cases were screened using electronic health records and discharge-diagnosis systems and confirmed by chart review. TBM diagnostic certainty was retrospectively harmonized according to the Marais uniform research case definition. The primary five-component neurological complication burden score included hydrocephalus, documented acute seizures, cerebral infarction, cerebral hemorrhage, and elevated intracranial pressure (ICP). Components could be present at admission or first documented later during the index hospitalization. Among non-survivors, chart review confirmed that no burden-score neurological complication was first documented within the final 48 h before death. Logistic regression and exploratory model-performance analyses were performed using complete-case data.

**Results:**

Among 1,574 non-HIV TBM patients, including 211 children/adolescents (≤18 years) and 1,363 adults (>18 years), 187 (11.9%) died during hospitalization. Mortality increased across primary five-component burden categories: 8.8% for 0 components, 13.3% for 1 component, 20.3% for 2 components, and 34.5% for ≥3 components. In adjusted models, documented acute seizures [adjusted odds ratio [aOR] 4.10; 95% confidence interval [CI], 2.43–6.90], hydrocephalus (aOR 3.06; 95% CI, 1.92–4.88), and elevated ICP (aOR 1.48; 95% CI, 1.03–2.11; *P* = 0.032) were independently associated with mortality. Adding neurological burden to the base model improved discrimination modestly (area under the curve from 0.596 to 0.652; DeLong *P* = 0.002), with acceptable calibration; exploratory decision curve analysis showed generally small between-model differences in net benefit.

**Conclusions:**

Cumulative neurological complication burden encompassing components present at admission or documented later during hospitalization was associated with stepwise increases in in-hospital mortality among non-HIV TBM patients across the lifespan. Hydrocephalus, documented acute seizures, and elevated ICP were key mortality-associated factors. The score should be interpreted as a study-developed summary of hospitalization-period neurological severity rather than as a baseline prediction model, causal exposure, or externally validated prognostic score.

## Introduction

TBM is a severe form of extrapulmonary tuberculosis and remains associated with substantial mortality and neurological disability. Adult systematic reviews have reported mortality of approximately 23%–25%, with substantial neurological sequelae among survivors (1, 2). Reported outcomes vary according to HIV status, disease stage, and cohort characteristics (1, 2). Microbiological confirmation rates and mortality estimates also vary substantially across study settings (3). In childhood TBM, pooled mortality was 19.3%, and 53.9% of survivors had neurological sequelae (4). These data support a lifespan perspective while recognizing age-related differences in presentation and outcome (5).

The high case-fatality rate reflects the pathophysiology of TBM, in which inflammatory exudates within the subarachnoid space initiate interconnected cerebrospinal fluid-flow, vascular, and parenchymal injuries (6). Hydrocephalus, cerebral infarction, seizures, and intracranial hypertension are clinically consequential complications linked to adverse outcomes or neurological deterioration (7–11). Because these complications may coexist or emerge sequentially, a cumulative summary may capture aspects of neurological severity not represented by isolated binary diagnoses.

Prior prognostic research has generally followed two approaches. Cohort studies and meta-analyses have evaluated individual neurological complications and conventional severity indicators (2, 7–11), whereas prognostic models have combined predominantly admission-based variables such as age, disease stage, neurological examination findings, cerebrospinal fluid indices, and selected imaging findings to predict later mortality or functional outcome (12–14). Anderson et al. described multiple neurological and systemic complications within a hospital cohort (15). To our knowledge, previous studies have not operationalized a simple equal-weight burden of neurological complications documented across one hospitalization or assessed its incremental contribution to a basic clinical model in a large non-HIV cohort spanning pediatric and adult patients.

We therefore conducted a large retrospective cohort study of non-HIV TBM patients across the lifespan to evaluate whether cumulative neurological complication burden was associated with all-cause in-hospital mortality. We also assessed whether adding this burden measure to a simple clinical model improved discrimination and calibration and explored its net-benefit characteristics across prespecified threshold probabilities.

## Materials and methods

### Study population

We conducted a single-center retrospective observational cohort study of patients diagnosed with TBM at Changsha Central Hospital between October 1, 2013, and January 1, 2024. Potentially eligible admissions were screened from the hospital electronic health record and discharge-diagnosis systems using ICD-10 diagnosis codes/terms corresponding to tuberculous meningitis, tuberculous cerebrospinal meningitis, and tuberculous encephalitis. Final inclusion was based on electronic chart review rather than ICD-10 coding alone, with confirmation against predefined eligibility criteria. TBM diagnostic certainty was retrospectively harmonized according to the uniform research case definition proposed by Marais et al. (16, which incorporates clinical, CSF, neuroimaging, and evidence-of-tuberculosis-elsewhere criteria, together with exclusion of alternative diagnoses. Definite TBM required microbiological or histopathological confirmation of central nervous system Mycobacterium tuberculosis infection. When neuroimaging was available, probable TBM required a diagnostic score of ≥12 and possible TBM a score of 6–11; when neuroimaging was unavailable, the corresponding thresholds were ≥10 and 6–9, respectively.

TBM diagnostic certainty was retained as an adjustment covariate in regression models. HIV-positive patients and patients with concomitant intracranial infections or insufficient key clinical data were excluded.

The study was approved by the Ethics Committee of Changsha Central Hospital (Approval No. 20250122). Given the retrospective design and use of anonymized data, the requirement for written informed consent was waived. The study was designed and reported in accordance with the Strengthening the Reporting of Observational Studies in Epidemiology (STROBE) guidelines (17).

### Analytic framework and terminology

The cohort included children/adolescents and adults because the primary objective was to estimate the overall association between neurological complication burden and mortality within one hospital system rather than to derive an age-specific prediction rule. This approach was informed by shared TBM pathophysiology across the lifespan (5), while age-related heterogeneity was addressed through age adjustment and descriptive age-stratified analyses. Throughout this report, “hospitalization-documented neurological complication burden” indicates the cumulative presence of qualifying complications that were already present at admission or were first documented later during the index hospitalization; it does not imply that all complications originated after admission. “Risk stratification during hospitalization” refers to contemporaneous severity assessment using information accumulated during the index stay, not prediction from a fixed admission baseline.

### Data collection and Variable definitions

Demographic characteristics, comorbidities, laboratory findings, CSF indices, neurological complications, and neuroimaging data were extracted from electronic medical records. The primary outcome was in-hospital mortality, defined as death from any cause during the index TBM hospitalization. The index date was the date of first hospitalization for TBM, and follow-up was restricted to the inpatient period.

Neurological complication variables were defined as physician-documented or imaging-documented features recorded during the index hospitalization rather than as fixed admission exposures. The term “hospitalization-documented” included complications already present at admission, complications that developed after admission, and complications first identified through clinical observation, ICP measurement, cerebrospinal fluid assessment, CT, or MRI during hospitalization. The exact timing relative to admission could not be reliably assigned for every component. Among non-survivors, chart review confirmed that no burden-score neurological complication was first documented within the final 48 h before death. The 48-hour window was selected pragmatically to distinguish complications recorded before the immediate dying process while preserving sufficient sample size. This rule reduced concern about purely preterminal ascertainment but did not establish exact onset or temporal ordering for every component. The variables were therefore analyzed as hospitalization-period severity markers rather than as purely baseline predictors or time-updated causal exposures. Operational definitions and sources are summarized in Supplementary Table 2.

The primary neurological complication burden score comprised five prespecified binary components: hydrocephalus, documented acute seizures, cerebral infarction, cerebral hemorrhage, and elevated ICP. Hydrocephalus was defined by CT or MRI evidence of ventricular enlargement, with Evans index measured on available cranial imaging; an Evans index >0.30 supported the diagnosis of ventriculomegaly/hydrocephalus. Documented acute seizures referred to acute seizure events or seizure-related clinical documentation during the index hospitalization, not to a remote history of epilepsy. It was defined by physician-documented seizure activity, an inpatient seizure diagnosis or acute seizure-related documentation, or inpatient antiseizure treatment for an acute seizure event. Cerebral infarction and cerebral hemorrhage were defined by compatible neuroimaging findings recorded during hospitalization. Elevated ICP was defined as ICP at or above the 75th percentile of the study distribution (190 mmH2O). Each component contributed one point, yielding a cumulative score categorized as 0, 1, 2, and ≥3 for descriptive analysis. This equal-weight score was developed for the present study as an operational summary of cumulative neurological injury load and has not yet been externally validated. The equal-weight structure was chosen as a transparent, clinically interpretable summary rather than a data-derived weighted prediction score. To evaluate robustness, a sensitivity six-component burden score additionally including the complicated MRI pattern was also constructed.

Brain MRI was performed using a 1.5 T or 3.0 T scanner with conventional and contrast-enhanced sequences, including T1-weighted, T2-weighted, FLAIR, and post-contrast imaging. Images were independently reviewed by two senior radiologists blinded to clinical and outcome information. The operational imaging categories used in this study were informed by previously described TBM and central nervous system tuberculosis imaging patterns (18–21). The categories were defined as follows:

Normal: no relevant structural or signal abnormality. Parenchymal lesions: focal or diffuse abnormal parenchymal signals, including infarcts, tuberculomas, tuberculous abscess/cerebritis, or encephalitic lesions (18–20). Meningeal enhancement: abnormal leptomeningeal or basal cisternal enhancement after contrast administration (18–20). Complicated MRI pattern: a study-defined composite category denoting multiple or complex TBM-related abnormalities, including hydrocephalus, infarction, edema or mass effect around tuberculomas, vasculitic changes, and hemorrhage when present (18–20). Spinal involvement: abnormal spinal cord, nerve-root, or meningeal signal/enhancement indicating spinal extension of neurotuberculosis (21).

Because the complicated MRI pattern was a study-defined composite category rather than a single anatomical lesion, it was excluded from the primary neurological complication burden score to reduce potential double counting with hydrocephalus, infarction, and hemorrhage. It was evaluated separately as an imaging-pattern variable and in a sensitivity six-component score.

### Missing data assessment and handling

Before modeling, variable types and missingness were evaluated. Missingness was low: the highest rate among raw variables was 1.21% (19/1,574). The outcome and primary neurological complication burden score were available for all included patients; albumin had one missing value among variables used in adjusted modeling. Because missingness was minimal, primary analyses used complete-case methods. Multiple imputation was not performed, as complete-case retention for the main model comparison was 1,573/1,574 (>99.9%), and the expected benefit of imputation was negligible relative to the additional modeling assumptions it would introduce.

### Statistical analysis

Continuous variables were summarized as medians and interquartile ranges (IQRs) and compared between survivors and non-survivors using the Wilcoxon rank-sum test. Categorical variables were summarized as counts and percentages and compared using Fisher's exact or chi-square tests, as appropriate. These comparisons were interpreted descriptively, especially for variables documented during hospitalization rather than strictly before admission.

For individual neurological complications, adjusted odds ratios (aORs) and 95% confidence intervals (CIs) were estimated using separate multivariable logistic regression models. Each model included one neurological complication as the exposure of interest and was adjusted for age, sex, TBM diagnostic certainty, immunodeficiency, diabetes mellitus, albumin, CSF glucose, CSF protein, and CSF chloride.

Age was included as an adjustment covariate in all primary models, and mortality according to neurological burden was summarized descriptively within children/adolescents (≤18 years) and adults (>18 years). Because the pediatric subgroup and high-burden pediatric cells contained few events, formal age-group-by-burden interaction testing was considered underpowered and potentially unstable. The age-stratified results were therefore treated as exploratory and were not used to claim that the relative burden-mortality association was identical across age groups.

For incremental performance assessment, four nested models were evaluated: the base model, the base model plus the primary five-component neurological complication burden score, the expanded model, and the expanded model plus the primary five-component neurological complication burden score. The base model included age, sex, TBM diagnostic certainty, immunodeficiency, and diabetes mellitus. The expanded model additionally included albumin, CSF glucose, CSF protein, and CSF chloride.

Model performance was assessed using discrimination, calibration, and decision curve analysis (DCA). Discrimination was evaluated using the area under the receiver operating characteristic curve (AUC) with 95% CIs. Calibration was assessed using the calibration intercept, calibration slope, Brier score, and Hosmer-Lemeshow test. DCA compared the net benefit of the four models with treat-all and treat-none strategies across threshold probabilities from 1% to 30% (22). Because burden components were ascertained across hospitalization rather than at a uniform prediction time, all model-performance analyses were considered exploratory and were not intended to establish a prospective clinical prediction model or prospective clinical utility.

Sensitivity analyses evaluated an alternative six-component neurological complication burden score that additionally included the complicated MRI pattern. Statistical analyses were performed using R version 4.5.3. All tests were two-sided, and *P* < 0.05 was considered statistically significant.

## Results

### Patient characteristics and missing data

**Figure 1:**
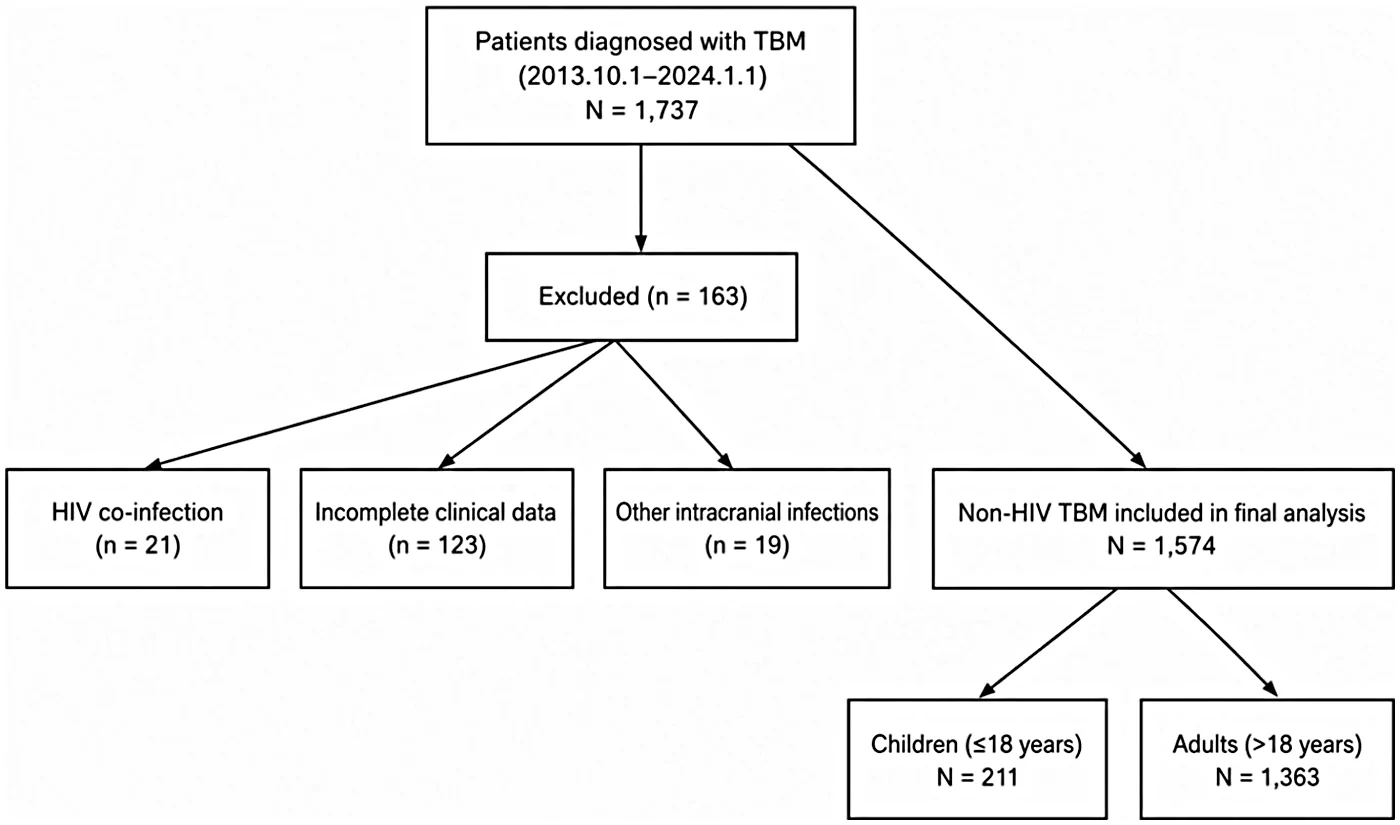
Flow diagram of patient selection.

Of 1,737 patients diagnosed with TBM between October 2013 and January 2024, 163 were excluded, including 21 with HIV co-infection, 123 with incomplete clinical data, and 19 with concomitant intracranial infections. The patient-selection process is shown in Figure 1. The final analytic cohort included 1,574 non-HIV TBM patients with available in-hospital survival status, comprising 211 children/adolescents (≤18 years) and 1,363 adults (>18 years). During hospitalization, 187 patients died and 1,387 survived, corresponding to an overall in-hospital mortality rate of 11.9%; mortality was 10.4% (22/211) among children/adolescents and 12.1% (165/1,363) among adults. Clinical, laboratory, imaging, and treatment characteristics stratified by survival status are shown in Table 1.

**Table 1:** Clinical characteristics and hospitalization-documented features stratified by outcome (survival vs. non-survival).

Variable	Overall	Survival	Non-survival	*P* value
	*N* = 1,574	*N* = 1,387	*N* = 187	
Demographics				
Age (years), median (IQR)	44.00 (25.00, 59.00)	44.00 (25.00, 59.00)	45.00 (28.00, 62.00)	0.055
Female, *n* (%)	1,035 (66%)	906 (65%)	129 (69%)	0.400
Comorbidities and clinical features				
Pulmonary tuberculosis type, *n* (%)				0.600
No pulmonary tuberculosis	347 (22%)	310 (22%)	37 (20%)	
Hematogenous dissemination	490 (31%)	422 (30%)	68 (36%)	
Primary pulmonary tuberculosis	53 (3.4%)	48 (3.5%)	5 (2.7%)	
Secondary pulmonary tuberculosis	679 (43%)	602 (43%)	77 (41%)	
Tuberculous pleurisy	5 (0.3%)	5 (0.4%)	0 (0%)	
Hydrocephalus, *n* (%)	119 (7.6%)	88 (6.3%)	31 (17%)	<0.001
Cerebral hemorrhage, *n* (%)	16 (1.0%)	11 (0.8%)	5 (2.7%)	0.033
Immunodeficiency, *n* (%)	452 (29%)	374 (27%)	78 (42%)	<0.001
Cerebral infarction, *n* (%)	265 (17%)	228 (16%)	37 (20%)	0.300
Documented acute seizures, *n* (%)	86 (5.5%)	61 (4.4%)	25 (13%)	<0.001
History of tuberculosis contact, *n* (%)	318 (20%)	284 (20%)	34 (18%)	0.500
Renal failure, *n* (%)	412 (26%)	334 (24%)	78 (42%)	<0.001
Laboratory findings				
Fibrinogen (g/L), median (IQR)	4.60 (2.83, 4.60)	4.60 (2.84, 4.60)	4.60 (2.83, 4.60)	0.200
Serum uric acid (*μ*mol/L), median (IQR)	370.50 (250.00, 524.00)	374.70 (250.00, 529.70)	296.00 (196.00, 515.00)	0.023
Hyponatremia, *n* (%)	442 (28%)	367 (26%)	75 (40%)	<0.001
Serum creatinine (μmol/L), median (IQR)	48.00 (36.00, 65.00)	47.00 (36.00, 63.00)	54.40 (36.00, 78.00)	<0.001
Positive CSF Mycobacterium tuberculosis culture, *n* (%)	30 (1.9%)	30 (2.2%)	0 (0%)	0.041
CSF white blood cell count ( × 10⁶/L), median (IQR)	10.00 (10.00, 60.00)	10.00 (10.00, 51.00)	11.00 (10.00, 100.00)	0.031
Proportion of mononuclear cells in CSF, median (IQR)	0.68 (0.50, 0.69)	0.68 (0.50, 0.69)	0.67 (0.50, 0.69)	0.044
CSF chloride (mmol/L), median (IQR)	124.20 (115.00, 125.00)	125.00 (116.00, 125.00)	121.00 (113.00, 125.00)	0.016
CSF glucose (mmol/L), median (IQR)	2.20 (2.20, 3.21)	2.20 (2.20, 3.21)	2.20 (1.93, 3.21)	0.200
CSF protein (g/L), median (IQR)	0.90 (0.59, 1.37)	0.90 (0.58, 1.32)	0.91 (0.60, 1.84)	0.013
CSF ADA (U/L), median (IQR)	2.43 (2.43, 3.00)	2.43 (2.43, 2.80)	2.43 (2.43, 5.00)	0.038
CSF Mycobacterium tuberculosis DNA detected, *n* (%)	45 (2.9%)	40 (2.9%)	5 (2.7%)	>0.900
Platelet count ( × 10⁹/L), median (IQR)	217.00 (154.00, 275.00)	217.00 (158.00, 275.00)	216.00 (125.00, 272.00)	0.052
ESR (mm/h), median (IQR)	20.00 (19.00, 39.00)	20.00 (18.00, 37.00)	20.00 (20.00, 46.00)	0.002
Hemoglobin (g/L), median (IQR)	114.15 (105.00, 126.00)	115.00 (105.00, 126.00)	110.00 (101.70, 119.00)	<0.001
Indirect bilirubin (μmol/L), median (IQR)	5.50 (3.30, 5.90)	5.50 (3.30, 5.86)	5.50 (3.42, 6.36)	0.500
PCT (ng/mL), median (IQR)	0.04 (0.03, 0.09)	0.04 (0.03, 0.08)	0.05 (0.03, 0.28)	<0.001
Albumin (g/L), median (IQR)	35.00 (32.00, 38.50)	35.00 (32.50, 38.80)	34.30 (29.00, 37.00)	<0.001
ICP (mmH2O), median (IQR)	150.00 (120.00, 190.00)	150.00 (125.00, 190.00)	150.00 (120.00, 220.00)	0.200
Drug-resistant tuberculosis, *n* (%)	98 (6.2%)	83 (6.0%)	15 (8.0%)	0.300
PPD positivity, *n* (%)	131 (8.3%)	114 (8.2%)	17 (9.1%)	0.700
Diagnostic tests				
CSF tuberculosis antibody positive, *n* (%)	195 (12%)	165 (12%)	30 (16%)	0.120
CSF acid-fast bacilli smear positive, *n* (%)	7 (0.4%)	6 (0.4%)	1 (0.5%)	0.600
Treatment				
Corticosteroid, *n* (%)	1,535 (98%)	1,351 (97%)	184 (98%)	0.600
Levofloxacin, *n* (%)	859 (55%)	758 (55%)	101 (54%)	0.900
Linezolid, *n* (%)	996 (63%)	884 (64%)	112 (60%)	0.300
Aspirin use, *n* (%)	844 (54%)	737 (53%)	107 (57%)	0.300
Brain MRI findings, *n* (%)				0.200
Normal	226 (14%)	205 (15%)	21 (11%)	
Parenchymal lesions	1,068 (68%)	939 (68%)	129 (69%)	
Meningeal enhancement	160 (10%)	140 (10%)	20 (11%)	
Complicated	81 (5.1%)	66 (4.8%)	15 (8.0%)	
Spinal involvement	39 (2.5%)	37 (2.7%)	2 (1.1%)	
TBM diagnostic category, *n* (%)				0.200
Definite	553 (35%)	493 (36%)	60 (32%)	
Probable	953 (61%)	830 (60%)	123 (66%)	
Possible	68 (4.3%)	64 (4.6%)	4 (2.1%)	

This table summarizes demographic, clinical, laboratory, diagnostic, imaging, and treatment characteristics of 1,574 non-HIV TBM patients stratified by outcome. Continuous variables are presented as median (IQR), and categorical variables as *n* (%). *P* values were derived from the Wilcoxon rank-sum test for continuous variables and the chi-square test or Fisher's exact test for categorical variables, as appropriate. Because some variables were hospitalization-documented and could have been present at admission or identified later during the index stay, comparisons are descriptive rather than purely pre-admission baseline contrasts. UA, uric acid; ALB, albumin; CSF, cerebrospinal fluid; AFB, acid-fast bacilli; PPD, purified protein derivative; PCT, procalcitonin; ESR, erythrocyte sedimentation rate; ICP, intracranial pressure; TBM, tuberculous meningitis; IQR, interquartile range.

### Neurological complication burden and mortality

**Figure 2:**
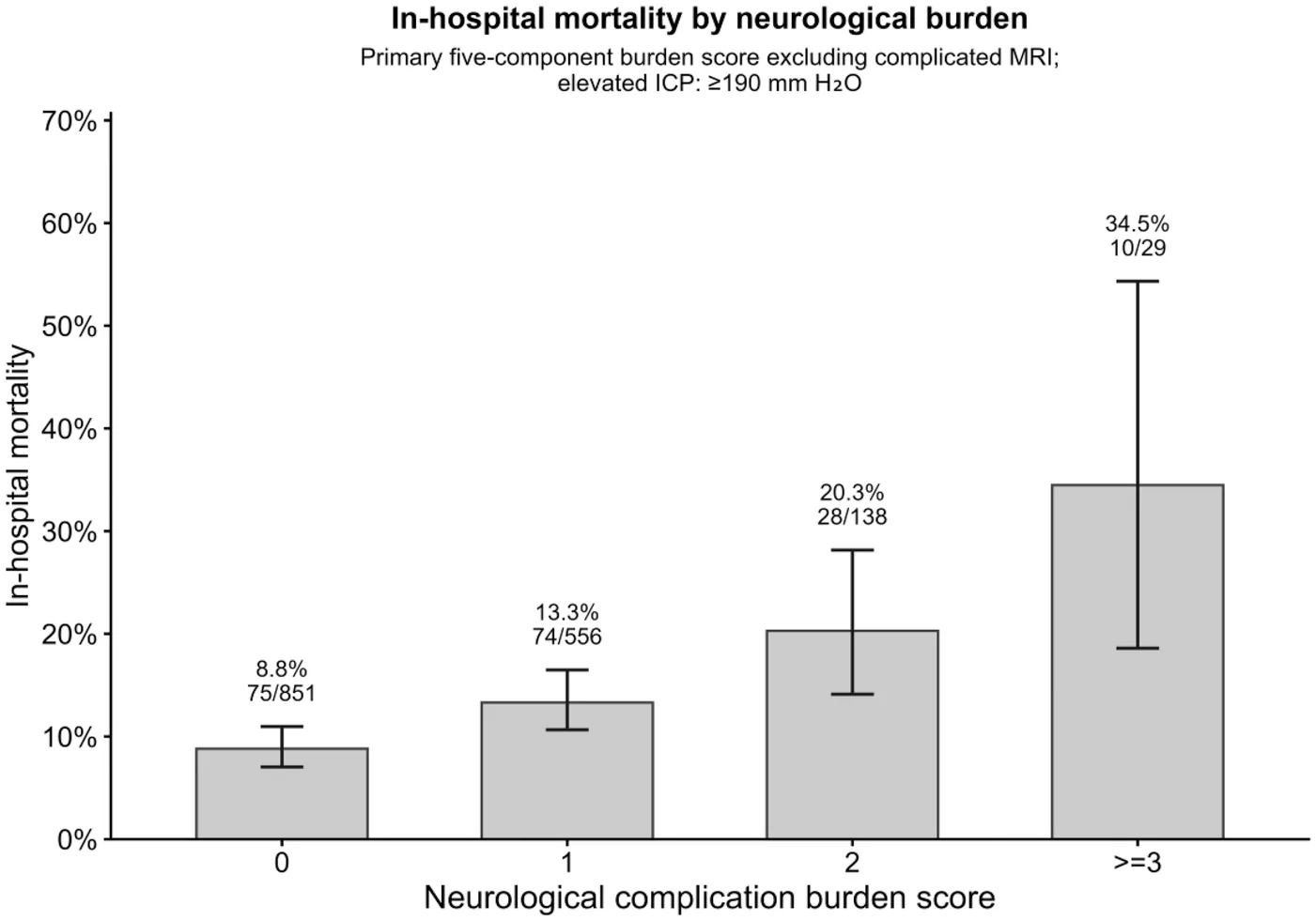
In-hospital mortality according to the primary five-component neurological complication burden score excluding the complicated MRI pattern. Elevated ICP was defined as ICP ≥75th percentile (190 mmH2O). Error bars indicate 95% confidence intervals. Denominators were 851, 556, 138, and 29 patients for 0, 1, 2, and ≥3 burden components, respectively; the ≥3 estimate is imprecise because of sparse data.

Using the prespecified terminal-phase handling rule, the primary five-component neurological complication burden score remained available for all 1,574 included patients. Among 187 non-survivors, no burden-score neurological complication was first documented within the final 48 h before death; all counted burden-score components had been documented earlier during hospitalization or at admission (Supplementary Table 3). Mortality increased in a graded pattern with higher burden categories: 8.8% (75/851) among patients with 0 components, 13.3% (74/556) with 1 component, 20.3% (28/138) with 2 components, and 34.5% (10/29) with ≥3 components (Figure 2). In descriptive age-stratified analyses, mortality increased across burden categories among adults (9.6%, 12.8%, 18.8%, and 45.5% for 0, 1, 2, and ≥3 components, respectively) and among children/adolescents through the 2-component category (4.1%, 17.7%, and 28.6% for 0, 1, and 2 components, respectively). No deaths occurred among the seven children/adolescents in the ≥3 category, making this subgroup estimate unstable rather than reassuring (Supplementary Table 1). In the sensitivity six-component score that additionally included the complicated MRI pattern, mortality was 8.5% (71/840), 14.2% (75/527), 17.3% (27/156), and 27.5% (14/51) across the corresponding burden categories (Supplementary Figure 1).

### Adjusted associations of individual neurological complications with mortality

**Figure 3:**
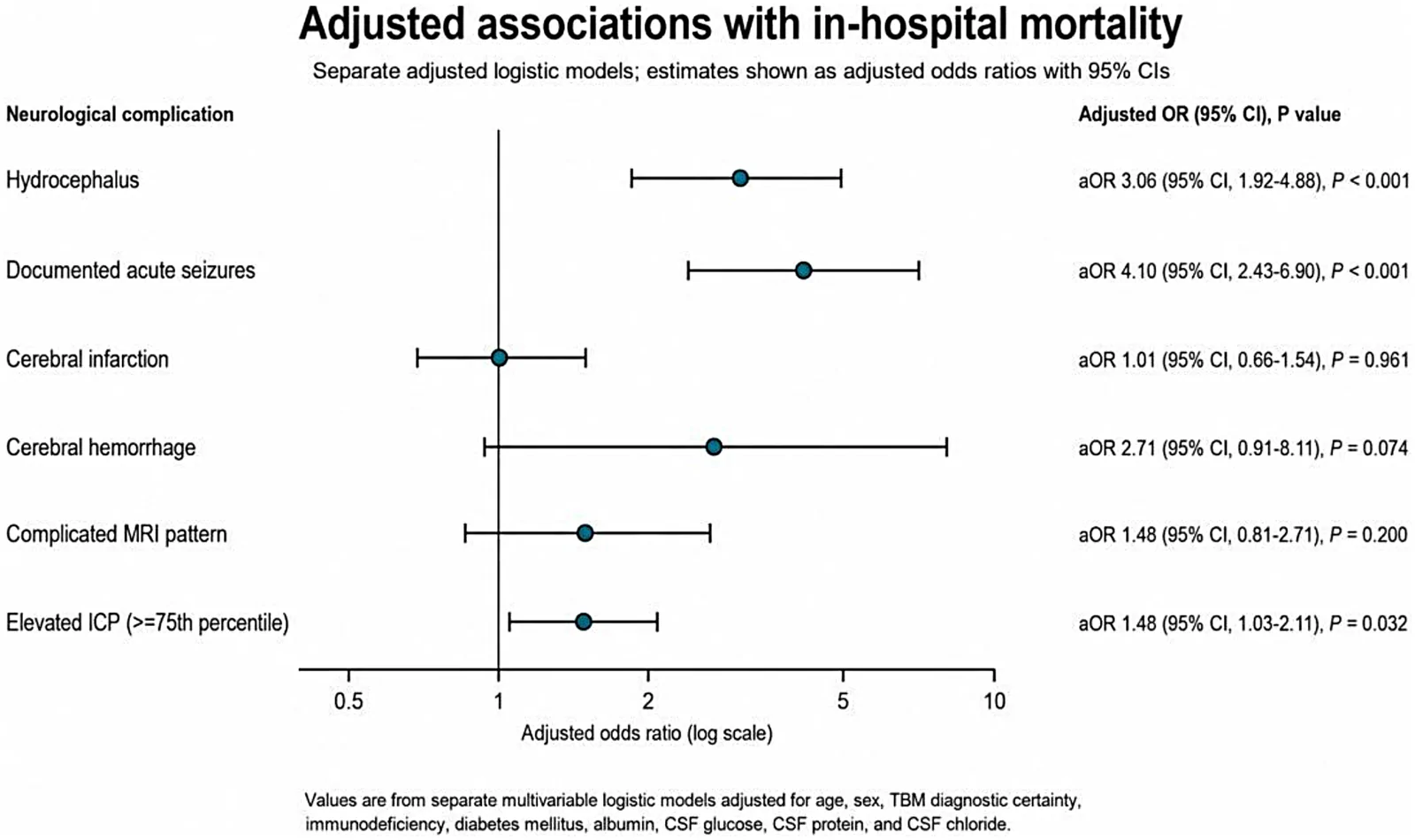
Adjusted associations between individual neurological complications and in-hospital mortality. Models were adjusted for age, sex, TBM diagnostic certainty, immunodeficiency, diabetes mellitus, albumin, CSF glucose, CSF protein, and CSF chloride. Elevated ICP was defined as ICP ≥75th percentile. Complicated MRI refers to the prespecified complicated MRI pattern.

In adjusted models, documented acute seizures showed the strongest association with in-hospital mortality (aOR 4.10; 95% CI, 2.43–6.90; *P* < 0.001), followed by hydrocephalus (aOR 3.06; 95% CI, 1.92–4.88; *P* < 0.001). Elevated ICP was also independently associated with mortality (aOR 1.48; 95% CI, 1.03–2.11; *P* = 0.032). Cerebral hemorrhage showed a directionally increased but statistically nonsignificant association (aOR 2.71; 95% CI, 0.91–8.11; *P* = 0.074). Neither the complicated MRI pattern nor cerebral infarction was independently associated with mortality after adjustment (Table 2; Figure 3).

**Table 2:** Adjusted associations between individual neurological complications and in-hospital mortality.

Neurological complication	Adjusted OR	95% CI low	95% CI high	*P* value
Hydrocephalus	3.06	1.92	4.88	<0.001
Documented acute seizures	4.10	2.43	6.90	<0.001
Cerebral infarction	1.01	0.66	1.54	0.961
Cerebral hemorrhage	2.71	0.91	8.11	0.074
Complicated MRI pattern	1.48	0.81	2.71	0.200
Elevated ICP, ≥75th percentile	1.48	1.03	2.11	0.032

Each neurological complication was fitted in a separate multivariable logistic model adjusted for age, sex, TBM diagnostic certainty, immunodeficiency, diabetes mellitus, albumin, CSF glucose, CSF protein, and CSF chloride. Components could have been present at admission or documented later during the index hospitalization and were not analyzed as fixed admission exposures. Among non-survivors, no burden-score component was first documented within 48 h before death. The complicated MRI pattern was analyzed separately and was not included in the primary five-component burden score. OR, odds ratio; CI, confidence interval; MRI, magnetic resonance imaging; ICP, intracranial pressure; TBM, tuberculous meningitis; CSF, cerebrospinal fluid.

### Exploratory incremental model performance and net benefit

**Figure 4:**
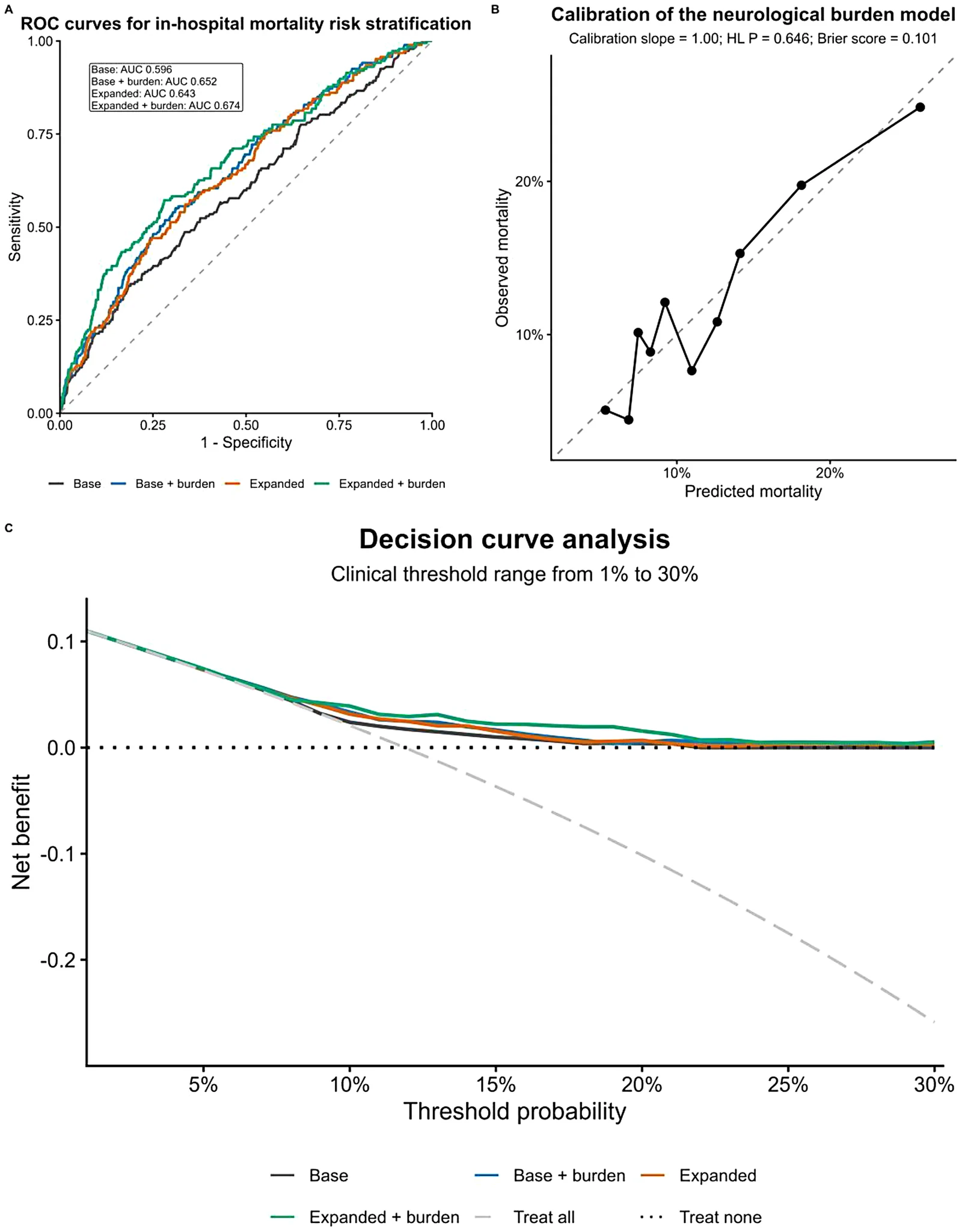
Exploratory model-performance assessment after adding the primary five-component neurological complication burden score. **(A)** Receiver operating characteristic curves for four models. **(B)** Calibration of the base model plus burden; the dashed line denotes ideal calibration. **(C)** Exploratory decision curve analysis across threshold probabilities from 1% to 30%. Because burden components were documented at nonuniform times, this analysis does not establish prospective clinical utility.

In the complete-case comparison (*N* = 1,573), the base model showed modest discrimination (AUC 0.596; 95% CI, 0.552–0.641). Adding the primary five-component neurological complication burden score improved discrimination (AUC 0.652; 95% CI, 0.611–0.694; DeLong *P* = 0.002; Figure 4A). The expanded model achieved an AUC of 0.643, and the expanded model plus neurological burden yielded the highest AUC of 0.674. Calibration of the base model plus neurological burden was acceptable (calibration slope 1.00; Brier score 0.101; Hosmer-Lemeshow *P* = 0.646; Figure 4B).

Exploratory DCA showed generally small differences in net benefit between models across the evaluated 1%–30% threshold-probability range (Figure 4C).

## Discussion

In this retrospective lifespan cohort of 1,574 non-HIV TBM patients, the primary five-component neurological complication burden documented across the index hospitalization was associated with a stepwise increase in in-hospital mortality. Patients with three or more components had a mortality rate of 34.5%, compared with 8.8% among patients without recorded components; the high-burden estimate was based on only 29 patients and was imprecise. Adding the burden score to the base model improved discrimination modestly. These findings support the score as a summary of hospitalization-period neurological severity, but they do not establish baseline prediction, causal effects, or benefit from score-guided management.

The present study extends prior TBM prognostic research in three respects. First, previous cohorts, systematic reviews, and prognostic models have mainly evaluated individual complications or admission-based demographic, clinical, laboratory, and imaging variables (2, 7–15). Second, integrating five neurological complications provides an operational framework for summarizing their clustering during one hospitalization. Third, inclusion of children/adolescents and adults allowed the overall burden-mortality pattern to be described within a single hospital system with age adjustment in the primary models. The pooled analysis does not eliminate age-related heterogeneity; age-stratified findings, particularly the sparse pediatric high-burden category, remain exploratory.

The association between cumulative neurological burden and mortality is biologically plausible because TBM complications are mechanistically interconnected. Inflammatory exudates within the subarachnoid space, particularly around the basal cisterns, can obstruct cerebrospinal fluid flow, promote hydrocephalus, increase ICP, and compromise cerebral perfusion (6, 11, 23). In parallel, TBM-related vasculitis and vascular occlusion may cause cerebral infarction, whereas meningeal irritation, tuberculomas, infarction, and metabolic abnormalities may contribute to seizures (6, 9, 11, 24). A cumulative measure may therefore summarize the combined severity of cerebrospinal fluid-flow, vascular, parenchymal, and seizure-related injury.

Among individual complications, documented acute seizures and hydrocephalus demonstrated the strongest independent associations with mortality, and elevated ICP was also independently associated with mortality. This pattern is consistent with evidence linking seizures to adverse outcomes in TBM (9, 24, 25), hydrocephalus to unfavorable prognosis (7, and raised ICP or impaired cerebral perfusion to neurological deterioration (11, 23). These associations do not prove that the complications directly caused death in individual patients; they identify clinically visible features of a severe neurocritical TBM phenotype.

Cerebral infarction was not independently associated with mortality in the adjusted model, although TBM-related stroke is common and is associated with substantial morbidity and mortality in prior studies (8, 26). A binary indicator does not capture vascular territory, lesion distribution, multiplicity, symptomatic status, or timing, all of which vary in TBM-related stroke (26, 27). The observed association may also be attenuated because infarction co-occurs with hydrocephalus, meningeal inflammation, and other disease-severity features (11, 27). The null adjusted estimate should not be interpreted as evidence that cerebral infarction is clinically unimportant. Future studies should use lesion-level and time-resolved measures.

The complicated MRI pattern was a study-defined composite imaging category comprising multiple or complex TBM-related abnormalities, including hydrocephalus, infarction, edema or mass effect around tuberculomas, vasculitic changes, and hemorrhage when present (18–20). Because these abnormalities arise through overlapping inflammatory, cerebrospinal fluid-flow, vascular, and parenchymal pathways (6), the category overlaps conceptually with specific binary complications. It was therefore excluded from the primary burden score and evaluated separately and in the six-component sensitivity analysis. Its null adjusted association may reflect within-category heterogeneity and overlap rather than absence of clinical relevance.

Adding the primary five-component burden score increased the AUC by 0.056, but overall discrimination remained moderate. Exploratory DCA showed generally small between-model differences in net benefit. Because the score incorporated information accumulated at nonuniform times during hospitalization, this analysis does not establish prospective clinical utility. The ≥3-component group contained 29 patients, producing a wide confidence interval around the 34.5% mortality estimate. As an author interpretation, the score may serve as a descriptive communication aid, but this potential use has not been prospectively evaluated. Independently of the score, established TBM neurocritical-care principles support neurological reassessment, ICP evaluation when indicated, neuroimaging review, hydrocephalus assessment, and prompt recognition and management of acute seizures (9, 11, 23). Whether score-guided use improves management or outcomes requires prospective evaluation, and the score should not be used as a stand-alone decision rule.

The pulmonary tuberculosis categories in Table 1 did not differ significantly between survivors and non-survivors (omnibus *P* = 0.600). Their crude distribution cannot establish a protective association, earlier recognition, or differential treatment response. The non-HIV setting reduces one major source of heterogeneity but limits generalizability because HIV-associated TBM differs in immune status, diagnostic and therapeutic challenges, and prognosis (28). Age was adjusted for in the primary models, and descriptive age-stratified results were reported without assuming identical relative associations across age groups. Non-HIV status also does not imply absence of immune vulnerability. Primary immunodeficiency has been described in HIV-negative TBM (29), and the present cohort included patients classified as immunodeficient or diabetic. These considerations support covariate adjustment and limit extrapolation to populations with different immunological profiles.

This study has several strengths. It is a large non-HIV TBM series spanning children/adolescents and adults and enabled estimation of overall complication-specific associations, although the estimate for rare cerebral hemorrhage remained imprecise. Individual and cumulative neurological complication phenotypes were examined within one analytic framework. The primary score excluded the composite MRI category, and a six-component sensitivity score assessed robustness to its inclusion. Missingness was minimal, and complete-case analysis retained 1,573 of 1,574 patients (>99.9%). Model evaluation included discrimination, calibration, and exploratory DCA. Chart review confirmed that burden-score complications were not first documented within the final 48 h before death. The study-defined imaging categories were informed by prior TBM imaging literature, and blinded radiological review reduced the risk of outcome-informed image interpretation.

Several limitations should be acknowledged. First, this was a single-center retrospective study using routinely collected electronic health record data; residual confounding, selection bias, and information bias cannot be excluded. Although ICD-10 codes and terms were used for screening, final eligibility required chart review and retrospective diagnostic classification. Second, complications were analyzed as hospitalization-documented features rather than time-updated covariates. Patients who survived longer may have had more opportunities for neurological examination, ICP measurement, CT, or MRI, whereas patients who died early may have had less opportunity for ascertainment. Third, the 48-hour rule reduced concern about immediate preterminal documentation but did not resolve exact onset or temporal ordering for every component; a landmark analysis was not feasible because onset and detection times could not be reconstructed consistently. Fourth, formal age-group interaction testing was not emphasized because pediatric high-burden cells were sparse. Fifth, the equal-weight score was developed for this study, and individual components differ in severity and prognostic importance. Sixth, model-performance estimates were apparent, in-sample estimates without resampling-based optimism correction or external validation, and DCA lacked a uniform prospective decision time. Seventh, lesion-level details such as infarct territory, distribution, and hemorrhage volume were unavailable. Finally, pulmonary tuberculosis categories and other hospitalization-documented features were analyzed descriptively and should not be interpreted causally. Independent cohorts with prospectively defined assessment times are needed to validate static and dynamic burden measures and to test whether their use improves patient outcomes.

## Conclusions

Cumulative neurological complication burden, encompassing components present at admission or documented later during the index hospitalization, was associated with stepwise increases in in-hospital mortality among non-HIV TBM patients across the lifespan. Hydrocephalus and documented acute seizures were the strongest individual mortality-associated factors, while elevated ICP was also independently associated with mortality. The burden score provided statistically significant but modest incremental discrimination. It should be interpreted as a study-developed summary of hospitalization-period neurological severity rather than as a baseline prediction model, causal exposure, externally validated prognostic score, or established clinical decision tool.

## Data Availability

The original contributions presented in the study are included in the article/Supplementary Material, further inquiries can be directed to the corresponding author.
