# Supplementary material for: Neurological complication burden and in-hospital mortality in non-HIV tuberculous meningitis across the lifespan: a retrospective cohort study
Source: Front Med (Lausanne). 2026 Sep 9;13:1895708. doi: 10.3389/fmed.2026.1895708 (PMC13597350; doi:10.3389/fmed.2026.1895708)
Supplement: Supplementary file 1 [file Table1.docx]

**Supplementary Appendix**

Supplementary Figure 1. In-hospital mortality according to the sensitivity six-component neurological complication burden score including complicated MRI pattern. Elevated ICP was defined as ICP ≥75th percentile. Error bars indicate 95% confidence intervals.

Supplementary Table 1. Descriptive age-stratified in-hospital mortality according to the primary five-component neurological complication burden score.

| **Age group** | **Burden components** | **Patients, n** | **Deaths, n** | **Mortality, %** |
| --- | --- | --- | --- | --- |
| Children/adolescents (≤18 years) | 0 | 121 | 5 | 4.1 |
| Children/adolescents (≤18 years) | 1 | 62 | 11 | 17.7 |
| Children/adolescents (≤18 years) | 2 | 21 | 6 | 28.6 |
| Children/adolescents (≤18 years) | ≥3 | 7 | 0 | 0.0 |
| Adults (>18 years) | 0 | 730 | 70 | 9.6 |
| Adults (>18 years) | 1 | 494 | 63 | 12.8 |
| Adults (>18 years) | 2 | 117 | 22 | 18.8 |
| Adults (>18 years) | ≥3 | 22 | 10 | 45.5 |

Note: These analyses are descriptive and should not be interpreted as formal evidence of age-invariant relative effects because pediatric high-burden cells were sparse.

Supplementary Table 2. Operational definitions and source/origin of diagnostic, complication, and burden-score variables.

| **Variable/framework** | **Operational definition** | **Source/origin** |
| --- | --- | --- |
| TBM diagnostic certainty | Definite, probable, or possible TBM retrospectively harmonized from clinical, CSF, imaging, microbiological, extracranial TB, and treatment-response information. | Marais 2010 uniform research case definition |
| ICD-10/EHR case screening | Potentially eligible admissions screened using ICD-10 diagnosis codes/terms for tuberculous meningitis, tuberculous cerebrospinal meningitis, and tuberculous encephalitis; final inclusion confirmed by electronic chart review. | Study case-ascertainment workflow |
| Final 48-hour terminal phase | The final 48 hours before in-hospital death. The window was selected pragmatically to separate events documented during the immediate dying process from complications recorded earlier during hospitalization. | Prespecified study rule |
| Five-component burden score | Hydrocephalus, documented acute seizures, cerebral infarction, cerebral hemorrhage, and elevated ICP; each component contributed 1 point. | Study-developed equal-weight operational score |
| Hydrocephalus | Radiology-reported or reviewer-confirmed ventricular enlargement on CT or MRI; when axial images allowed measurement, Evans index >0.30 was used as supportive radiological evidence. | Imaging-based definition |
| Documented acute seizures | The database variable originally labeled 'Epilepsy' was redefined for this analysis as documented acute seizures during the index TBM hospitalization, including physician-documented acute seizure activity, in-hospital seizure diagnosis or acute seizure-related documentation, or inpatient antiseizure treatment for an acute seizure event. | Study operational definition |
| Cerebral infarction | Compatible neuroimaging evidence of cerebral infarction recorded during hospitalization. | Imaging-based definition |
| Cerebral hemorrhage | Compatible neuroimaging evidence of cerebral hemorrhage recorded during hospitalization. | Imaging-based definition |
| Elevated ICP | ICP at or above the 75th percentile of the study distribution (190 mmH2O). | Study distribution-based threshold |
| Complicated MRI pattern | Multiple TBM-related imaging abnormalities, including hydrocephalus, infarction, edema or mass effect around tuberculomas, vasculitic changes, and hemorrhage when present. | Adapted from TBM neuroradiological literature |

Supplementary Table 3. Neurological complications first documented within the final 48 hours before death among non-survivors.

| **Complication** | **First documented within final 48 hours before death, n/N (%)** |
| --- | --- |
| Hydrocephalus | 0/187 (0.0) |
| Documented acute seizures | 0/187 (0.0) |
| Cerebral infarction | 0/187 (0.0) |
| Cerebral hemorrhage | 0/187 (0.0) |
| Elevated ICP | 0/187 (0.0) |
| Any burden-score neurological complication | 0/187 (0.0) |

Note: The denominator is the 187 non-survivors. Chart review confirmed that no burden-score neurological complication was first documented within the final 48 hours before death.
